# P-916. Use of BCID2 on CSF in nosocomial CNS surgical site infections

**DOI:** 10.1093/ofid/ofae631.1107

**Published:** 2025-01-29

**Authors:** Laura Walters, Devin Weber, Jamie Nassur, Matthew Pettengill

**Affiliations:** Thomas Jefferson University Hospital, Glenside, Pennsylvania; Department of Medicine, Division of Infectious Diseases, Sidney Kimmel Medical College at Thomas Jefferson University, Philadelphia, PA; Sidney Kimmel Medical College, Thomas Jefferson University, Philadelphia, Pennsylvania; Department of Clinical Laboratories, Thomas Jefferson University Hospitals, Philadelphia, PA

## Abstract

**Background:**

Nosocomial intracranial infections, arising from surgical procedures or neuroinvasive devices like ventricular drains or shunts, present distinct risk factors and microbiologic etiologies compared to community-acquired meningitides. Pathogens typically include Staphylococcus spp., Gram-negative bacilli, and multidrug-resistant bacteria. The current diagnostic standard for nosocomial central nervous system (CNS) infections is cerebrospinal fluid (CSF) Gram stain and culture, but rapid molecular diagnostics could improve diagnosis and treatment, enhancing patient outcomes. This pilot study aimed to assess the validity of the BioFire® Blood Culture Identification Panel 2 (BCID2) panel used for blood culture positivity detection on CSF samples in patients presenting with nosocomial meningitis/ ventriculitis. Sensitivity and specificity were calculated, and patient characteristics and co-morbidities were reviewed.

Microbiologic data comparing gold standard Gram stain and culture of CSF to BCID2 result.

**Figure:**
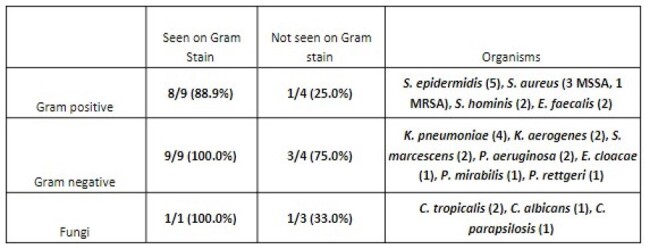

**Methods:**

Patients over 18, admitted to Thomas Jefferson University Health System hospitals between 4/1/2017 to 4/1/2024, were included if meeting Centers for Disease Control (CDC) criteria for intracranial infection or meningitis/ventriculitis.

Positive CSF samples (n=30) from 28 patients with nosocomial infections included Gram-positive bacteria (13), Gram-negative bacteria (13), and fungi (4). Positive CSF samples included those positive on culture, while control samples (n=26) had negative cultures or cultures positive for Cutibacterium acnes, an organism not detected by the BCID2 panel.

**Results:**

Across all positive CSF specimens, sensitivity for organism detection was 76.7%. When culture-positive samples were classified based on Gram Stain, samples with organisms detected on Gram Stain had a sensitivity of 94.7% compared to 45.5% for samples with no organisms seen. For samples with organisms detected on Gram Stain, sensitivity for Gram-negative organisms was 100% and 88.9% for Gram-positive organisms. Among all control specimens, specificity was 100%.

**Conclusion:**

The ability to rapidly identify a pathogen is crucial for managing nosocomial CNS infections effectively. Our study found that the BCID2 panel offers a promising utility in the detection of nosocomial CNS infections through CSF analysis.

**Disclosures:**

**Matthew Pettengill, PhD**, Cepheid: Grant/Research Support

